# Effects of DMSO on the Pluripotency of Cultured Mouse Embryonic Stem Cells (mESCs)

**DOI:** 10.1155/2020/8835353

**Published:** 2020-10-15

**Authors:** Maria Inês Sousa, Bibiana Correia, Ana Filipa Branco, Ana Sofia Rodrigues, João Ramalho-Santos

**Affiliations:** ^1^Department of Life Sciences, University of Coimbra, Calçada Martim de Freitas, 3000-456 Coimbra, Portugal; ^2^CNC-Center for Neuroscience and Cell Biology, CIBB, Azinhaga de Santa Comba, University of Coimbra, Coimbra, Portugal

## Abstract

DMSO is a commonly used solvent in biological studies, as it is an amphipathic molecule soluble in both aqueous and organic media. For that reason, it is the vehicle of choice for several water-insoluble substances used in research. At the molecular and cellular level, DMSO is a hydrogen-bound disrupter, an intercellular electrical uncoupler, and a cryoprotectant, among other properties. Importantly, DMSO often has overlooked side effects. In stem cell research, the literature is scarce, but there are reports on the effect of DMSO in human embryoid body differentiation and on human pluripotent stem cell priming towards differentiation, via modulation of cell cycle. However, in mouse embryonic stem cell (mESC) culture, there is almost no available information. Taking into consideration the almost ubiquitous use of DMSO in experiments involving mESCs, we aimed to understand the effect of very low doses of DMSO (0.0001%-0.2%), usually used to introduce pharmacological inhibitors/modulators, in mESCs cultured in two different media (2i and FBS-based media). Our results show that in the E14Tg2a mESC line used in this study, even the smallest concentration of DMSO had minor effects on the total number of cells in serum-cultured mESCs. However, these effects could not be explained by alterations in cell cycle or apoptosis. Furthermore, DMSO did not affect pluripotency or differentiation potential. All things considered, and although control experiments should be carried out in each cell line that is used, it is reasonable to conclude that DMSO at the concentrations used here has a minimal effect on this particular mESC line.

## 1. Introduction

Dimethyl sulfoxide (DMSO) is a commonly used aprotic solvent, which is soluble in both aqueous and inorganic media due to its polar domain and two apolar groups, enabling it to dissolve a wide range of small molecules [1–3]. It is therefore one of the most frequent solvents used in biological studies, as a vehicle for drug therapy.

However, DMSO is also a hydrogen-bound disrupter, an intercellular electrical uncoupler, and a hydroxyl radical scavenger, among other properties [1, 3], and could conceivably affect cell culture, depending on the cell type and the concentration used, issues that are usually overlooked. For instance, it has already been shown that apart from inducing cytotoxicity [4–6], DMSO can affect cell cycle [6–10] and mitochondrial function [5, 11], as well as the epigenetic landscape of different cell types [12–14], and influence the differentiation of various types of stem cells [7, 15–18]. Indeed, in 2012, Pal and colleagues reported that when exposing differentiating human embryonic stem cells (hESCs) to three different concentrations of DMSO, there was a dose-dependent downregulation of pluripotency markers and that the lowest concentration biased the differentiation process by enhancing mesodermal differentiation [17]. A similar effect was also observed in differentiating mouse stem cells from embryonal carcinoma [8]. More importantly, it was also reported that pretreatment of hESC and human-induced pluripotent stem cells (hiPSCs) with 1-2% (v/v) DMSO, for 24 hours prior to differentiation induction, improved their responsiveness and competency for differentiation into multiple lineages [7] by increasing the percentage of cells in the G1 phase of the cell cycle through the activation of the retinoblastoma pathway [7, 19].

Altogether, despite the scarce literature on the topic, it is clear that DMSO can affect the status of pluripotent stem cell cultures. However, little is known on the effect of DMSO on mESC culture. In fact, only recently, it was shown that DMSO treatment (0.1%-2%) for four days could sustain mESC pluripotency in the absence of LIF [20]. This evidence highlights the importance of evaluating the impact of smaller concentrations of DMSO in overall mESC culture status, particularly considering the diversity of studies using DMSO as a solvent for many compounds applied to mESC culture.

In this study and following initial observations that suggested a possible effect of DMSO present in the medium as a vehicle for several experimental compounds, we aimed to more systematically understand the effect of very low doses (0.0001%-0.2%), usually used to introduce pharmacological inhibitors/modulators, on mESC culture proliferation, pluripotency status, and differentiation potential.

## 2. Materials and Methods

### 2.1. mESC Culture and Experimental Design

**Figure 1:**
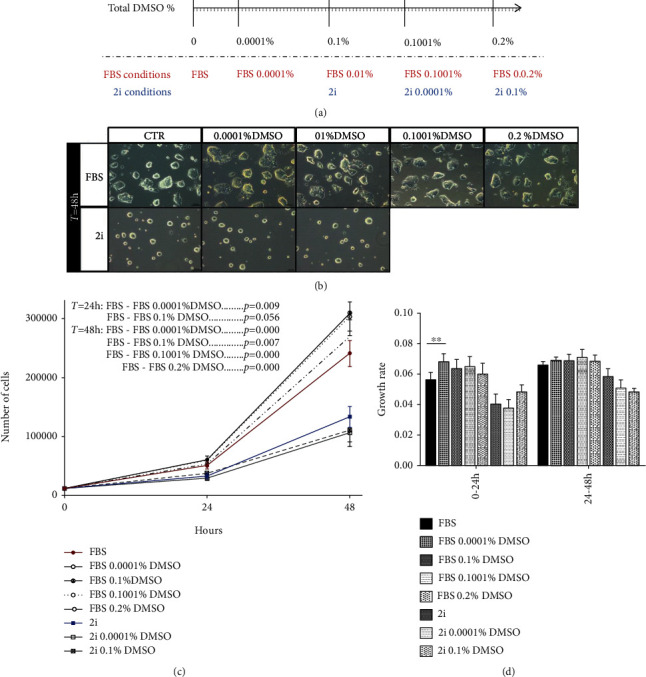
A small amount of DMSO has minimal effects in mESC culture. mESCs were cultured in two different media that mimic the naive pluripotency state and were kept in culture for 48 hours in the presence or absence of DMSO (control conditions—2i, FBS; DMSO-incubated conditions—0.0001% DMSO, 0.1% DMSO, 0.1001% DMSO, and 0.2% DMSO). (a) Schematic representation of the DMSO conditions used. (b) Representative images of plated cells acquired from randomly selected fields using phase contrast microscopy at the 48 h time point (100x magnification). (c) Total number of cells per well, 24 and 48 hours after treatment, from at least five independent experiments. (d) Daily growth rate analysis calculated using the total number of cells counted in each time point. Results are expressed as means ± SEM. Statistical significance considered when ^∗^*p* ≤ 0.05, ^∗∗^*p* ≤ 0.01, and ^∗∗∗^*p* ≤ 0.001.

Mouse embryonic stem cell line E14Tg2a was cultured in two different media that promote the naïve pluripotent stem cell state: a homogeneous naïve state (2i medium) and a heterogeneous naïve state (FBS medium) at 37°C and 5% CO_2_ conditions. 2i medium consisted of a 1 : 1 solution of DMEM/F12 (Gibco, Thermo Fisher Scientific) and Neurobasal medium (Gibco, Thermo Fisher Scientific), supplemented with 100 U/ml penicillin/streptomycin (Gibco, Thermo Fisher Scientific), 2 mM L-glutamine (Gibco, Thermo Fisher Scientific), 0.1 mM 2-mercaptoethanol (Sigma-Aldrich), 1 : 200 N2 and 1 : 100 B27 (Gibco, Thermo Fisher Scientific), 1x Leukemia inhibitory factor (LIF), and MEK/GS3 Inhibitor Supplement from the ESGRO®-2i Supplement Kit (Merck). The serum-based medium consisted of DMEM (Gibco, Thermo Fisher Scientific), supplemented with 15% embryonic stem cell-qualified FBS (Gibco, Thermo Fisher Scientific), 100 U/ml penicillin/streptomycin, 0.1 mM 2-mercaptoethanol, 1% nonessential amino acids (Sigma-Aldrich), 2 mM sodium pyruvate (Thermo Fisher Scientific), and 1x10^6^ U/ml of LIF (Merck). When culture reached optimal confluence, cells were passage using the dissociation reagent StemPro™ Accutase™ Cell Dissociation Reagent (Merck-Millipore) and plated in previously 0.1% gelatin-coated (Sigma-Aldrich) plates at a 8000 cell/cm^2^ density. To assess the effect of DMSO (Sigma-Aldrich) on mESC culture, 2i- and FBS-cultured mESCs were exposed to different concentrations of DMSO, for up to 48 hours. The concentrations tested in this study were chosen having in consideration the most common dilutions used to introduce pharmacological inhibitors/modulators in biological research: from two consecutive 1 : 1000 dilutions (1 : 10^6^)–0.0001% to the regular 1 : 1000 dilution–0.1% and other combinations mimicking the addition of two compounds each diluted in 1 : 10^6^ or/and 1 : 1000-0.1001% and 0.2%. In order to prevent possible pipetting errors, intermediate stock solutions were prepared whenever necessary and subsequently diluted in culture medium. It is also important to note that the cells cultured in the 2i medium are already adapted to 0.1% of DMSO, which is the vehicle for the 2i inhibitors already present. In our experimental design, we tried to mimic what would happen if more DMSO had to be added at different time points (due to the addition of other compounds), which is a common occurrence in this type of experiments (see Figure 1(a)).

### 2.2. Image Acquisition and Cell Count

Image acquisition and cell count experiments were performed using a phase-contrast microscope (Leica DMI3000B) with a 10X objective and a Leica DFC425C camera. To measure culture growth, cells were plated at an 8000 cells/cm^2^ density in a 48-well plate and two replicates of every condition were performed in each independent experiment (*n*). After 24 and 48 hours of incubation with the different concentrations of DMSO, colonies were dissociated with Accutase for 5 minutes at 37°C. The enzyme was inactivated by dilution with fresh medium, and a small aliquot was used for Trypan Blue staining (Sigma-Aldrich) at a 1 : 1 ratio. Cell count was performed with a hemocytometer, and total number of cells per well was calculated [21, 22]. Growth rate was then calculated using the mathematical formula—growth rate = (ln(*x* + 24h) − ln(*x*))/24h, were *x* stands for the total number of cells in one time point and *x* + 24 h stands for the total number of cells counted 24 hours after *x*.

### 2.3. Embryoid Body (EB) Differentiation

To evaluate if the differentiation process was being affected by the pretreatment of mESCs with DMSO, the embryoid bodie (EB) suspension protocol [23] was performed. For this purpose, 10^6^ mESCs of each condition were plated in nonadherent 60 mm Petri dish and maintained in suspension for three days in FBS medium or 2i medium without LIF supplementation. The medium was changed on the second day of culture in suspension using the EB sedimentation technique [23]. After three days in suspension, EBs were plated in a gelatin-coated 100 mm tissue-cultured Petri dish to allow adherence in a FBS-medium supplemented with a final serum percentage of 25% or in a mixture of basal DMEM- F12 and Neurobasal medium supplemented with 100 U/mL penicillin/streptomycin and 25% FBS. Medium was changed every day, and RNA and protein were extracted at day 7 for the FBS cultured EBs or day 13 for the 2i cultured EBs.

### 2.4. Immunocytochemistry

The immunocytochemistry protocol was performed as previously described [22, 24]. In summary, cells were rinsed with warm PBS, fixed with 4% paraformaldehyde (Sigma-Aldrich) for 15 minutes at room temperature, rinsed three times with PBS, permeabilized with methanol at -20° C for 5 minutes, rinsed three more times with PBS, and blocked with a 5% BSA (Sigma-Aldrich) and 0.3% Triton X-100 (Sigma-Aldrich) PBS solution at room temperature. Then, they were incubated overnight at 4°C in an antibody solution consisting of 1% BSA and 0.3% Triton X-100 PBS. The primary antibodies used in this study were anti-Nanog (Cat. No. 80892; Abcam), anti-Smooth Muscle Actin (SMA; Cat. No. ab7817; Abcam), anti-*α*-Fetoprotein (*α*-FP; Cat. No. #3903; Cell Signaling Technology), and anti-*β*-3-tubulin (*β*-3-TUB; Cat. No. T5076; Sigma-Aldrich). Cells were then rinsed three times with PBS and incubated for 1 hour at room temperature with the antibody solution containing the respective secondary antibody (Alexa Fluor 488 or Alexa Fluor 568; Thermo Fisher Scientific). Nuclear staining was achieved by a 10 min incubation with Hoechst 33342 (Sigma-Aldrich) and three last PBS rinses, before image acquisition.

### 2.5. Flow Cytometry Protocols for Apoptosis/Necrosis and Cell Cycle Analysis

Flow cytometry was performed to evaluate alterations in cell cycle profile and apoptosis/necrosis [22, 23, 25]. Samples were analyzed in a Becton Dickinson BD FACSCalibur cytometer, and 20,000 cells were gated per condition and evaluated with the Cell Quest Pro Acquisition software (BD Biosciences).

For cell cycle analysis, cells were harvested 24 and 48 hours after treatment with DMSO. The cell suspension was then washed with warm PBS, fixed in ice-cold 70% ethanol and kept at -20° C overnight. The next day, cells were incubated with 0.1 mg/ml RNase A (Life Technologies) and 5 *μ*g/ml propidium iodide (PI) (Life Technologies) at room temperature for 15 min, centrifuged, resuspended in PBS, and analyzed by flow cytometry using the appropriate settings.

Evaluation of apoptosis and necrosis was performed using the Annexin V (Immunostep) and PI protocol [22, 23] at the 24 and 48 hour time points. After harvesting, cells were centrifuged, washed once with PBS and 10^6^ cells/ml, per condition, were resuspended in Annexin V binding buffer (10 mM Hepes/NaOH (pH 7.4) 140 mM NaCl, and 2.5 mM CaCl2), and incubated in the dark for 15 minutes at room temperature with Annexin V (50 *μ*L/ml) and 2.5 *μ*g/ml PI. After staining, cells were analyzed by flow cytometry. The appropriate experiment settings were defined with a blank negative control (cells not stained with either PI or Annexin V) and two positive controls for apoptosis and cell death; in which cells were incubated overnight in 0.05% H_2_0_2_ (Sigma-Aldrich) and stained with PI and Annexing V separately. This allowed us to obtain three distinct populations (the population of unstained/live cells, the population of dead cells (only stained with PI), and the population of apoptotic cells (only stained with Annexin V)).

### 2.6. RNA Extraction, DNA Cleanup, cDNA Synthesis, and Quantitative Real-Time PCR

Total RNA isolation was performed using the NZYol reagent (Nzytech) standard protocol. In this protocol, NZYol was added to the pellet of harvested cells, and after disruption with a vortex, chloroform (Sigma-Aldrich) was added. Subsequently, the mixture was centrifuged to separate a lower green phenol-chloroform phase with an interphase from a colourless upper aqueous phase containing the RNA, which was then collected and precipitated with 2-propanol at a 1 : 1 ratio [22, 26, 27]. Possible genomic DNA contamination was eliminated using the DNA-free kit (Ambion, Invitrogen). RNA concentration and quality were assessed using a NanoDrop ND-1000 spectometer (Controltecnica). Samples presenting a 260/280 ratio under 1.85 were discarded. After quantification, one microgram of RNA was used to synthesize cDNA using the iScriptTM cDNA Synthesis Kit (Bio-Rad) in a C1000 Thermal Cycler (BioRad), according to manufacturer's instructions. qPCR was performed using mouse-specific primers obtained from a primer bank database (http://pga.mgh.harvard.edu/primerbank/). *Oct4*—forward primer (FP): CGGAAGAGAAAGCGAACTAGC/reverse primer (RP): ATTGGCGATGTGAGTGATCTG; *Nanog*—FP: TCTTCCTGGTCCCCACAGTTT/RP: GCAAGAATAGTTCTCGGGATGAA; *Rexo1*—FP: CCCTCGACAGACTGACCCTAA/RP: TCGGGGCTAATCTCACTTTCAT; *Essrb*—FP: GCACCTGGGCTCTAGTTGC/RP: TACAGTCCTCGTAGCTCTTGC; *Cdkn1a* (*p21*)*—*FP: AGAAGGTACTTACGGTGTGGT/RP: GAGAGATTTCCCGAATTGCAGT; *Trp53* (*p53*)—FP: GTCACAGCACATGACGGAGG/RP: TCTTCCAGATGCTCGGGATAC; *Actb*—FP: GGCTGTATTCCCCTCCATCG/RP: CCAGTTGGTAACAATGCCATGT. For each condition, a genomic contamination control was performed in which the RNA sample was not converted in cDNA by the reverse transcriptase enzyme and was submitted to a PCR reaction. If a negative control was amplified by the PCR reaction, an additional step of DNA contamination elimination was executed. SsoFast EvaGreen Supermix (Bio-Rad) was used to perform RT-PCR reaction, and analysis was done according to Bio-Rad instructions in a CFX96 Touch Real-Time PC Detection System. The obtained qPCR data was analyzed recurring to double delta Ct method.

### 2.7. Protein Extraction, Quantification, and Western Blot

Protein extraction for Western blot was performed as previously described [22, 25, 26]. Cells were lysed in RIPA buffer (Sigma-Aldrich) supplemented with 2 mM of phenylmethylsulphonyl fluoride-PMSF (Sigma-Aldrich), 2x Halt phosphatase inhibitor cocktail (Pierce, Rockford, IL), and 0.2% protease inhibitor cocktail CLAP (Sigma-Aldrich). For protein quantification, the Pierce™ BCA (Bicinchoninic Acid) Protein Assay Kit was used according to the manufacturers' indications and absorbance was measured by a BioTek Synergy HT multidetection microplate reader (BioTek Instruments). Two replicates of each sample and calibration curve point were performed. Samples were denatured by diluting 30 *μ*g of protein in Laemmli sample buffer (Bio-Rad) in a 1 : 1 ratio and heating the mixture in a dry-bath at 95°C for 5 minutes. Afterwards, samples were loaded into a 12% Acrilamide Tris-HCl gel, and electrophoresis was performed in a Mini protean tetra cell Bio-Rad apparatus for protein separation. Proteins were then blotted onto a PDVF membrane (Bio-Rad), blocked in a 5% powder milk or 5% BSA (Bio-Rad) Tris-Buffered Saline with Tween (TBS-T) solution, depending on the primary antibody to be used for 1 h, and incubated overnight at 4° C with the following antibodies: anti-Oct4 (Cat. No. 701756; Thermo Fisher Scientific), anti-Nanog; anti-SMA, anti-*α*-FP, anti-*β*-3-TUB, anti-P53 (Cat. No. #2524; Cell Signaling Technology), and anti-*β*-actin (Cat. No. A2228; Sigma-Aldrich). Then, membranes were washed with TBS-T and incubated for 1 h at room temperature with the appropriate HRP-conjugated secondary antibody (Cell Signaling Technology). Clarity Western ECL Substrate (Bio-Rad) was used for protein detection in a VersaDoc Imaging system (BioRad). To analyze and quantify the results, the Quantity One (BioRad) software was used. Results were normalized for the respective *β*-actin bands.

### 2.8. Data Analysis and Statistics

For statistical analysis, the SPSS Statistics 20.0 (SPSS Inc.) software was used. All results are presented as mean ± SEM for ≥3 independent experiments. The raw data was verified for normality and homogeneity of variance, and the appropriated parametric (one-way ANOVA and two-tailed paired sample *t*-test) or nonparametric tests (Kruskal-Wallis and Mann-Whitney) were applied accordingly. Statistical significance was determined at ^∗^*p* ≤ 0.05, ^∗∗^*p* ≤ 0.01, and ^∗∗∗^*p* ≤ 0.001.

## 3. Results

### 3.1. The Addition of a Small Percentage of DMSO Can Affect mESC Culture

DMSO is one of the most common solvents used as a carrier vehicle for diverse compounds in biological studies. However, DMSO itself can have side effects that are usually overlooked, possibly influencing the effects of the inhibitors/modulators that are used in these studies. In stem cell research, especially in mESC culture, the literature is scarce, but there are some reports on the effect of DMSO in human embryoid body differentiation and on human pluripotent stem cell priming towards differentiation. In fact, it has already been shown that pretreatment of human pluripotent stem cells with DMSO primed the culture for differentiation [7, 19]. Nevertheless, in these studies, the percentage of DMSO used was around 1%, which is a much higher concentration than the percentage of DMSO used in the majority of published studies. Therefore, we aimed to evaluate the effect of very low relevant concentrations of DMSO on mESC culture proliferation, pluripotency status, and differentiation potential. For this purpose, we exposed naïve E14Tg2a mESCs cultured in two different media (a homogeneous naïve state—2i medium and a heterogeneous naïve state—FBS medium) to two or four different concentrations of DMSO for 48 hours. These concentrations were chosen considering the most common dilutions used to introduce pharmacological inhibitors/modulators in this type of research: from two consecutive 1 : 1000 dilutions (1 : 10^6^)–0.0001% to the regular 1 : 1000 dilution–0.1% and other combinations mimicking the addition of two compounds each diluted in 1 : 10^6^ or/and 1 : 1000-0.1001% and 0.2%. Considering that the basic 2i medium already contains 0.1% DMSO and thus the 2i-cultured mESCs are already adapted to this percentage of DMSO, we were interested in evaluating if the response to the addition of more DMSO in 2i-cultured mESCs (which is common in many experimental designs) would have a different effect from the addition of the same total percentage of DMSO in FBS-cultured mESCs (that are not accustomed to DMSO). Thus, we determined the range of concentrations of DMSO according to the predicted total final percentage of DMSO in the culture media (Figure 1(a)).

After 48 hours in the presence of DMSO, cells cultured in both media presented the same phenotype than the cells from the control conditions, with normal-sized round birefringent colonies with well-defined borders corresponding to a pluripotent phenotype (Figure 1(b)). Importantly, in the FBS-cultured conditions that represent a more heterogeneous naïve mESC culture, we did not observe an increase in the amount of spontaneously differentiating colonies (Figure 1(b)). To evaluate the effect of DMSO on mESC proliferation, we performed a growth curve assay (Figure 1(c)), and our results revealed that in the 2i-cultured cells, there were no significant differences on the total number of cells in culture (after 24 and 48 hours). However, the total number of FBS-cultured mESCs obtained after incubation with the smallest percentage of DMSO tested was significantly higher after 24 hours. Moreover, after 48 hours of incubation, almost all of the DMSO-treated conditions presented a significantly higher number of cells in culture when compared to the control (Figure 1(c)). However, this increase did not translate in a significant increase in culture growth rate, as there was only one significant difference observed between the control and the 0.0001% of DMSO at the 24 hour time point (Figure 1(d)). These results suggest an effect of DMSO on serum-based cultured E14Tg2a mESCs possibly related to an imbalance between apoptosis and proliferation.

### 3.2. DMSO Does Not Affect the Apoptotic and Cell Cycle Profiles of Cultured mESCs

**Figure 2:**
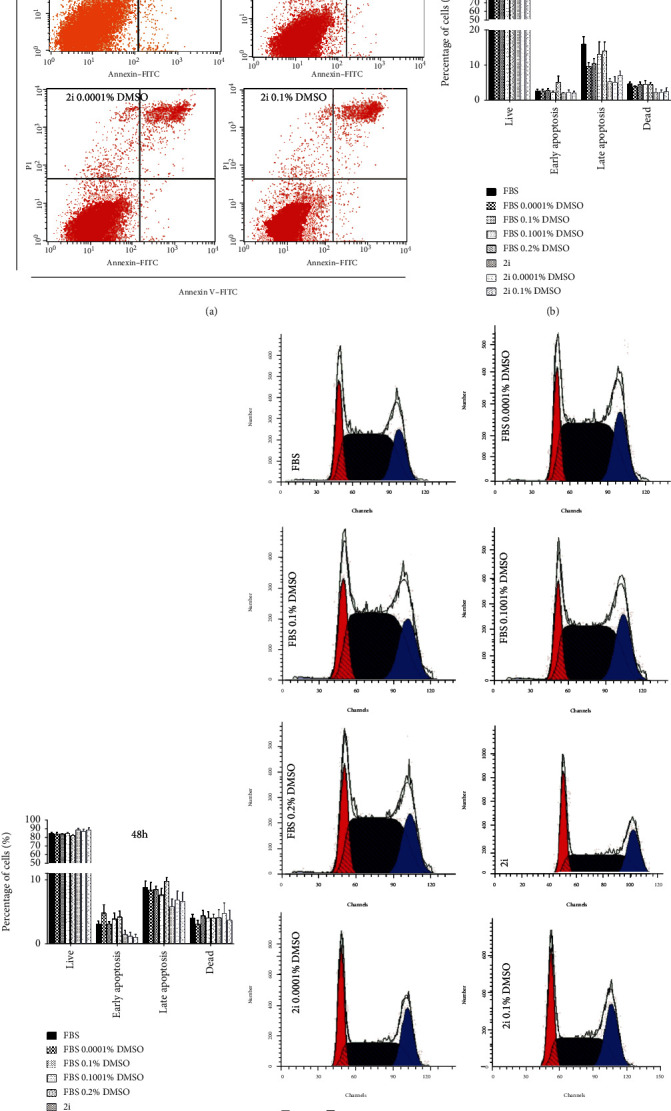
DMSO does not affect apoptotic and cell cycle profiles in cultured mESCs. The percentage of apoptotic/necrotic cells was assessed via Annexin V and PI staining after 24 and 48 h culture in the presence/absence of DMSO. (a) Flow cytometry dot plot from necrosis/apoptosis detection at the 48 h time point. Four populations were identified: live cells (negative for both Annexin V and PI), early apoptotic cells (positive for Annexin V and negative for PI), late apoptotic cells (positive for both Annexin V and PI), and dead cells (only positive for PI). (b) Percentage of cells in the defined populations at the 24 h time point. (c) Percentage of cells in the defined populations at the 48 h time point. Four independent experiments were conducted. (d) Flow cytometry histograms obtained from the analysis of cell cycle using PI staining at the 48 h time point. (e) Percentage of cells in each cell cycle phase at the 24 h time point. (f) Percentage of cells in each cell cycle phase at the 48 h time point. At least five independent experiments were performed. Results are expressed as means ± SEM. Statistical significance was considered when ^∗^*p* ≤ 0.05.

Due to the previously observed effects of DMSO on the total number of serum-cultured mESCs, we pondered if DMSO was having a prosurvival effect on mESCs (by reducing apoptosis) and evaluated the percentage of apoptotic/necrotic cells in culture by flow-cytometry. Our results show that exposure to DMSO for 24 hours does not affect the apoptotic/necrotic status of the mESC culture. Interestingly, in the FBS-cultured mESCs, a nonsignificant minor decrease in the percentage of late apoptotic/necrotic cells in the 0.0001% and 0.1% DMSO conditions was observed when compared to the control (Figures 2(a) and 2(b)). Additionally, after 48 hours of incubation with DMSO, there were no observable differences between the apoptotic profiles of the DMSO-treated conditions and the controls in both mESCs cultured in 2i and FBS media (Figures 2(a) and 2(c)). To complement our analysis, we evaluated the possible effects of DMSO in the cell cycle profile of cultured mESCs (Figure 2(d)). Interestingly, both the controls and the DMSO-treated conditions displayed identical cell cycle profiles after 24 and 48 hours in culture (Figures 2(d)–2(f)). There were also no significant differences in the expression levels of the cell cycle modulators analyzed (Supplementary Figure 1). Overall, we can conclude that adding of small percentage of DMSO in this mESC line culture for up to 48 hours does not significantly impact mESC culture proliferation, nor does it induces apoptosis.

### 3.3. Neither Pluripotency or Differentiation Potential of mESC IS Affected by Pretreatment with Low Concentrations of DMSO

**Figure 3:**
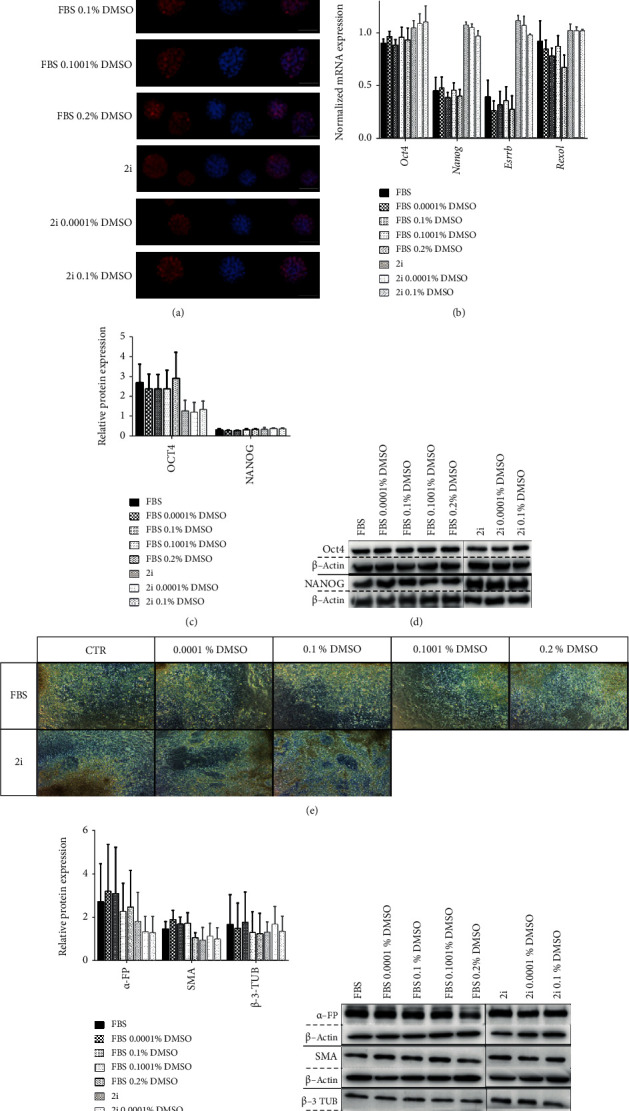
Exposure to small percentages of DMSO for 48 hours does not negatively affect pluripotency marker expression or the differentiation potential of mESC. (a) Representative immunofluorescence images of plated colonies presenting the pluripotency marker Nanog (red) and counterstained with Hoechst 33342 (blue) (600x magnification) after a 48 h incubation with/without DMSO. (b) RT-PCR analysis for *Oct4*, *Nanog*, *Rexo1*, and *Essrb* mRNA gene expression normalized for endogenous beta-actin (*Actb*) at the 48 h time point. At least four independent experiments were performed, and the results are presented as means ± SEM. (c, d) Relative protein expression of the pluripotency factors Oct4 and Nanog evaluated by Western blot analysis and normalized by *β*-actin expression at the 48 h time point. At least three independent experiments were performed, and the results are presented as means ± SEM. (e–g) Embryoid bodies (EBs) were generated from mESCs cultured for 48 h in the presence/absence of DMSO. After the differentiation protocol, every condition was able to generate fully differentiated cultures as shown in (e) by phase-contrast microscopy (100x magnification). (f, g) Western blot analysis and quantification of the protein levels of the three embryonic leaflet markers *α*-FP, SMA, and *β*-3-TUB normalized by the expression of the loading control *β*-actin. At least four independent experiments were performed, and the results are presented as means ± SEM. Statistical significance was considered when ^∗^*p* < 0.05.

To assess if the exposure of mESCs to very small doses of DMSO affected their pluripotency status, we evaluated the expression of hallmark genes of pluripotency. After 48 hours of exposure to DMSO, in both 2i- and FBS-cultured ESCs, the controls and the treated conditions presented the typical Nanog nuclear staining (Figure 3(a)) and showed identical levels of expression of the evaluated pluripotency genes (Figure 3(b)) that translated in similar protein levels of Oct4 and Nanog (Figures 3(c) and 3(d)). Therefore, we can assume that these small amounts of DMSO in mESC culture media did not affect overall pluripotency status. To corroborate these results, we evaluated the effects of DMSO on the differentiation potential of the mESC cell lines by performing the *in vitro* embryoid body assay and analyzing the expression of differentiation markers representative of the three embryonic leaflets. Because the FBS mESC culture is more heterogeneous, they differentiate easily; hence, it only took 7 days to obtain fully differentiated cultures, whilst when using 2i-cultured cells, it took approximately 13 days to reach the same state (Figure 3(e), Supplementary Figure 2A). According to our observations, pretreatment of both 2i- and FBS-cultured mESCs with small doses of DMSO for 48 hours before the induction of differentiation did not influence the expected differentiation timeline. Furthermore, DMSO-treated conditions presented the same protein levels of the differentiation markers analyzed when compared to the controls (Figures 3(f) and 3(g); Supplementary Figure 2B, 2C, D), which indicates that the exposure of mESCs to these small concentrations of DMSO did not influence the differentiation potential of cultured mESC. Taken together, these results confirm that the pluripotency status of E14Tg2a mESCs in culture is not affected by a 48-hour period of incubation with DMSO.

## 4. Discussion

DMSO is an aprotic solvent commonly used as a vehicle for drug therapy in biological studies. However, DMSO may have side effects on cell culture that are usually overlooked. For example, it has already been shown that DMSO can affect cell cycle [6–10], mitochondrial function [5, 11], and the epigenetic landscape of various cell types [12–14]. More importantly, DMSO can prime the differentiation of various types of stem cells [7, 15–18], and it is able to sustain mESC culture pluripotency in the absence of LIF [20]. However, in most of these studies, the range of DMSO concentrations used is not broad and is usually close to 1%, which is a much higher concentration than the amount of DMSO used in the majority of the *in vitro* biological studies published. For that reason, in this study, we aimed to evaluate the effect of smaller, realistically used, amounts of DMSO in the proliferation and pluripotency status of mESC culture. Thus, we exposed naïve E14Tg2a mESCs cultured in two different media to two or four different concentrations of DMSO for 48 hours (ranging from 0.0001% to 0.2% DMSO) considering the total percentage of DMSO predicted to in the final culture media. Unexpectedly, we detected an increase in the total number of cells in FBS-cultured mESCs with the lowest concentration of DMSO that translated in an increased growth rate after 24 h. Additionally, after a 48 h exposure, almost all the concentrations of DMSO induced a significant increase in the total number of cells in culture, but only in FBS-cultured mESCs. This difference in response to the addition of DMSO between the 2i- and FBS-cultured conditions could be due to the fact that the 2i-cultured mESCs are already adapted to the presence of DMSO in the medium. However, the underlying mechanisms responsible for this effect are still unknown. It is possible, since DMSO can affect the plasma membrane permeability of some cell types [28, 29], that in our study model, it could be affecting the cell membrane permeability to certain components of the FBS culture medium, allowing for enhanced culture survival when compared to the 2i-cultured mESCs.

Nonetheless, the observed increase in total number of cells in the FBS-cultured mESCs did not translate into an increase in growth rate, which suggests that these small amounts of DMSO could be modulating either apoptosis or proliferation. In fact, it has already been shown that exposure of human pluripotent stem cells to 1-2% of DMSO almost doubled the percentage of cells in the G1 phase [7]. Furthermore, DMSO-induced differentiation of various types of pluripotent and cancer cells was associated with a G1 cell cycle arrest and a decrease in c-myc levels [8, 30, 31]. Nevertheless, our results indicate that the concentrations of DMSO used were not sufficient to induce any alterations on the cell cycle profile in mESC cultures. We also hypothesized that DMSO could be having an effect on apoptosis as it was previously reported that DMSO could not only affect cell cycle progression, but also inhibit apoptosis in differentiating cancer cells [32]. However, despite a nonsignificant minor decrease in the percentage of late apoptotic/necrotic cells after 24 h in the 0.0001% and 0.1% DMSO conditions (that could partially explain the increase in total number of cells in culture), the overall results suggest that these small amounts of DMSO do not affect the percentage of apoptotic cells. We can therefore conclude that adding small amounts of DMSO to mESCs of this particular cell line for up to 48 hours does not significantly impact proliferation, nor does it affect apoptosis.

Since it has already been shown that DMSO is a promoter of differentiation and lineage commitment, it was of utmost importance to evaluate the pluripotency status and differentiation potential of our cultures when exposed to DMSO. According to the literature, DMSO is commonly used in the differentiation protocol of several cancer cell lines [30, 31, 33] and embryonic stem cell lines [8, 13, 16–18] in both mouse and human. In fact, recently several protocols have been established where DMSO pretreatment of human pluripotent cells is the key step to enhance efficiency of the differentiation protocol [19, 34]. Yet, our analysis showed that exposure to much smaller amounts of DMSO for 48 hours did not affect pluripotency status or differentiation potential. This is of great importance, because if these smaller amounts of DMSO promoted a bias in mESC differentiation, the results obtained in studies where a modulator/inhibitor was dissolved in DMSO could have been influenced by the presence of the vehicle.

All things considered, our results suggest that although mESC incubation with the most common amounts of DMSO used to introduce pharmacological inhibitors/modulators in biological research may have some minor effects on mESC culture, those effects are not sufficient to impact mESC cell cycle profile and apoptosis. Importantly, DMSO did not affect the pluripotency status of the culture nor did it influence the differentiation potential of these cells. Therefore, we can conclude that DMSO at very low concentrations normally used to add pharmacological agents has a minimal effect on E14Tg2a mESC culture, although we cannot rule out that line-specific effects may occur. Thus, further research on the effect of DMSO on other mESC cell lines is required.

## Data Availability

All relevant data is included in the manuscript. The authors will provide raw data upon request.

## Supplementary Materials

Supplementary Materials Supplementary Figure 1: it shows that DMSO does not influence the expression and presence of the apoptosis regulators p21 and p53 at both the RNA and protein level using qRT-PCR and Western blot, respectively. Supplementary Figure 2: it shows that DMSO does not influence the differentiation potential of mESCs using the embryoid body (EB) assay and immunocytochemistry using differentiation markers for the three germ layers: *α*-FP (endoderm), SMA (mesoderm), and *β*-3-TUB (ectoderm).
