# Supplementary material for: Effects of DMSO on the Pluripotency of Cultured Mouse Embryonic Stem Cells (mESCs)
Source: Stem Cells Int. 2020 Oct 15;2020:8835353. doi: 10.1155/2020/8835353 (PMC7584961; doi:10.1155/2020/8835353)
Supplement: Supplementary Materials — Supplementary Figure 1: it shows that DMSO does not influence the expression and presence of the apoptosis regulators p21 and p53 at both the RNA and protein level using qRT-PCR and Western blot, respectively. Supplementary Figure 2: it shows that DMSO does not influence the differentiation potential of mESCs using the embryoid body (EB) assay and immunocytochemistry using differentiation markers for the three germ layers: α-FP (endoderm), SMA (mesoderm), and β-3-TUB (ectoderm). [file 8835353.f1.doc]

Supplementary Material

**Supplementary Figures**

**
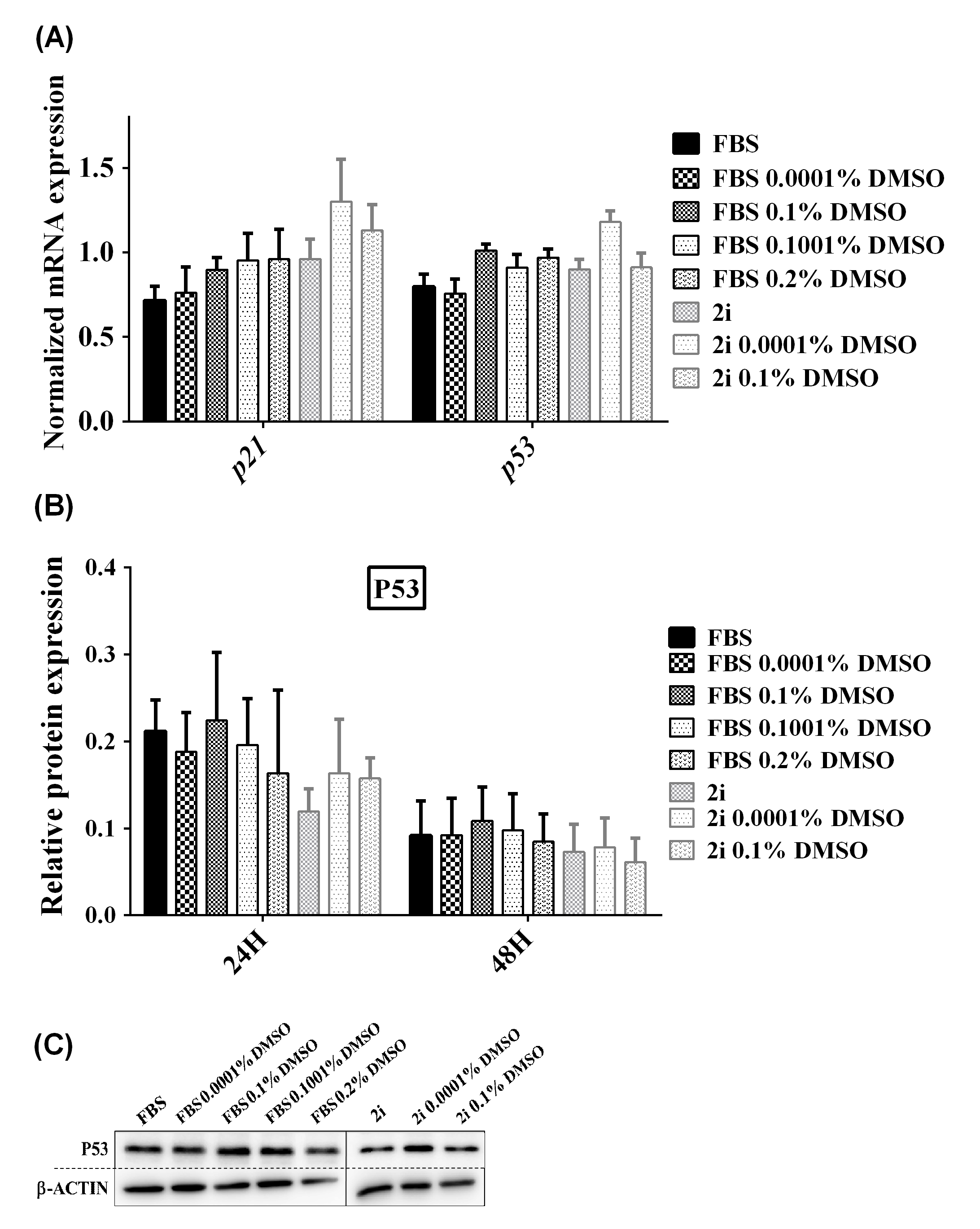
**

**Supplementary Figure 1.** Small amounts of DMSO do not affect expression of P53 and P21 in mESCs. **(A)** RT-PCR analysis for *p21* and *p53* gene expression, normalized for endogenous beta-actin (*Actb*), at the 48h time-point. At least four independent experiments were performed and results are presented as means ± SEM (**B, C**) Western-blot analysis and quantification of the expression of P53, after 24 and 48 h of incubation with DMSO, normalized by the expression of the loading control β-ACTIN. At least three independent experiments were performed and results are presented as means ± SEM.


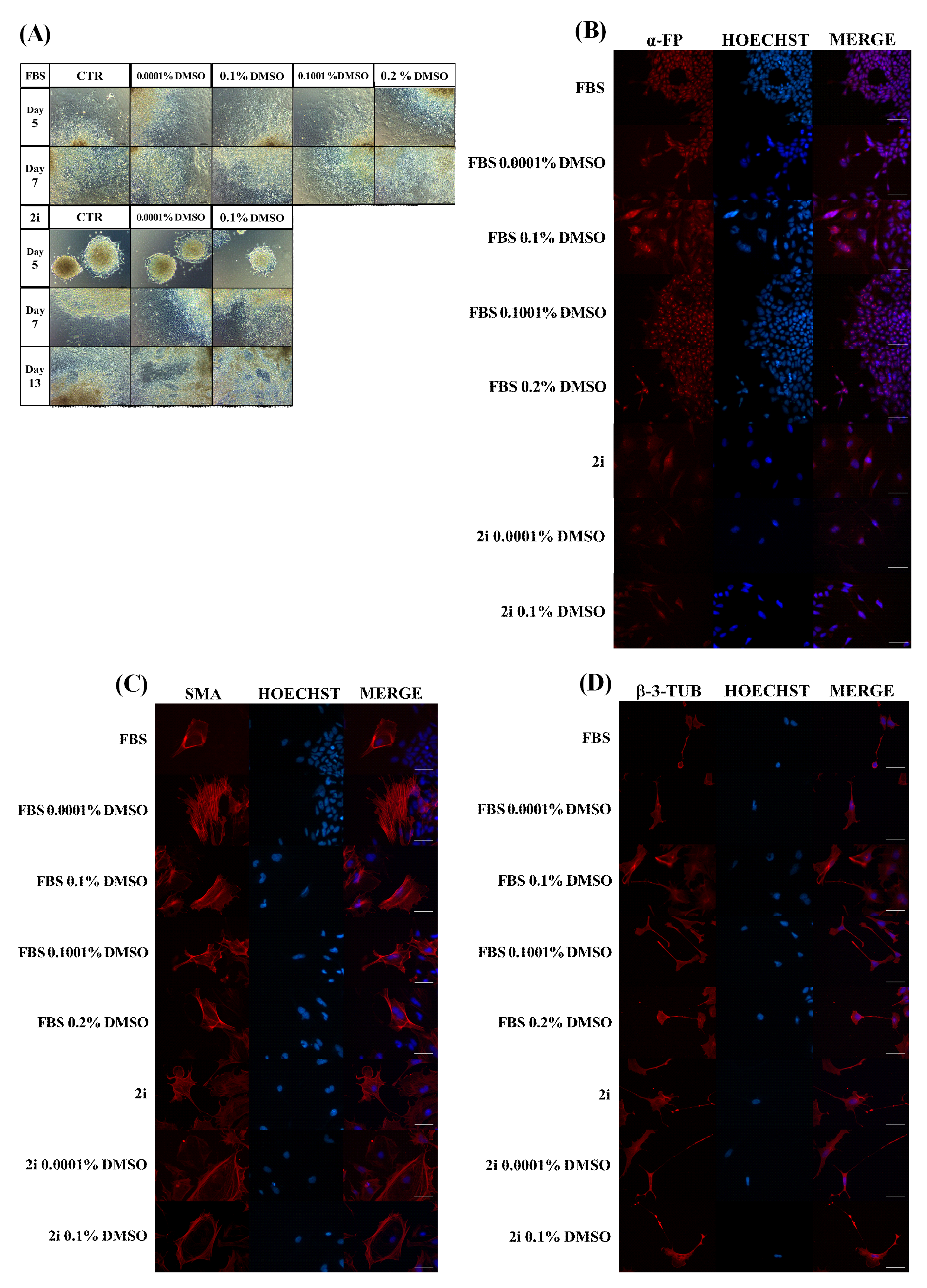


**Supplementary Figure 2.** DMSO does not affect mESC differentiation. **(A)** Progression of the embryoid bodies (EBs) assay. After 48 h in culture in the presence/absence of DMSO, 10^6^ mESCs of each condition were allowed to randomly differentiate. Every condition was able to generate fully differentiated cultures as shown by phase-contrast microscopy (100x magnification), after 7 days (for the FBS cultured cells) or 13 days (for the 2i cultured cells). **(B-D)** Representative immunofluorescence images acquired from randomly selected plated EBs, presenting three different markers of late differentiation: α-FP (endoderm), SMA (mesoderm) and β-3-TUB (ectoderm) (600x magnification).
